# Obesity and Obesogenic Behaviors in Asian American Children with Immigrant and US-Born Mothers

**DOI:** 10.3390/ijerph17051786

**Published:** 2020-03-10

**Authors:** Bianca R. Argueza, Karen Sokal-Gutierrez, Kristine A. Madsen

**Affiliations:** 1 California Department of Public Health Preventive Medicine Residency Program, Sacramento, CA 95899, USA; 2 Berkeley School of Public Health, University of California, Berkeley, CA 94720, USA; ksokalg@berkeley.edu (K.S.-G.); madsenk@berkeley.edu (K.A.M.)

**Keywords:** obesity, immigrants, nativity, Asian American, children

## Abstract

Child obesity is understudied in Asian Americans, which include a growing population of recent immigrants. We examined the relationship between maternal nativity and time in the US, and obesity and obesogenic behaviors among Asian American children. We analyzed public-use data from the 2013–2016 California Health Interview Survey for Asian American children ages 2 to 11 years. We used logistic regression to determine the odds of obesity and obesogenic behaviors associated with maternal nativity and time in the US. This study included n = 609 children. Children of US-born mothers had lower odds of obesity (adjusted odds ratio, AOR, 0.12; 95% CI 0.02 to 0.91) and lower fruit intake (AOR 0.15, 95% CI 0.03 to 0.81) than children of recent immigrants (< 5 years in the US). Asian American children with recent immigrant mothers are more likely to be obese and eat less fruit than children with US-born mothers. Efforts to prevent obesity and increase fruit consumption are particularly important for this vulnerable population of children of recent immigrants.

## 1. Introduction

Asian American children are under-investigated in obesity research. In 2015–2016, 23.2% of Asian American children in the United States (US) were overweight, and 10.7% were obese [1]. While the prevalence of overweight/obesity is relatively low in this population compared to other racial/ethnic groups [1,2,3], there is a need to better understand risk factors for elevated body mass index (BMI) in Asian American children for several reasons. First, some Asian American ethnic subgroups have a higher prevalence of overweight/obesity than others [4,5,6,7]. One study found that Filipino and Vietnamese American children are more likely to have elevated BMI than their white peers [7]. Second, the prevalence of elevated BMI is increasing in some Asian American children. The national prevalence of obesity among Asian American girls increased sharply from 5.6% in 2011–2012 to 10.1% in 2015–2016 [1]. Third, a substantial proportion of Asian individuals are at risk of cardiometabolic disease at BMIs below the threshold for overweight [8], so the relatively low prevalence of overweight/obesity in this population may not be reassuring. Finally, Asian immigrants comprise the largest proportion of new arrivals to the US [9], and Asian Americans are the fastest growing racial/ethnic group overall [10]. With the growing Asian American population, it is increasingly important to examine the health of this group, particularly among recent immigrants.

One factor that may affect child BMI is maternal acculturation. As immigrants settle in the US, they may adopt Western obesogenic behaviors through acculturation, a complex process through which individuals from one culture adopt the practices of another [5,11]. Given the role of maternal influence on dietary [12,13,14,15] and sedentary behaviors [16,17,18], mothers may pass obesogenic habits onto their children and increase their obesity risk. Exploring how acculturation might influence obesity and obesogenic behaviors may identify opportunities for intervention.

### 1.1. Maternal Acculturation and BMI

The small body of literature exploring the relationship between maternal acculturation and BMI in Asian American children is mixed. Maternal acculturation, as measured by English proficiency, has been shown to be a risk factor for consistent overweight/obesity in early elementary school children with less educated mothers but a protective factor in those with more educated mothers [5]. Another study found that Asian American preschool-age children with immigrant mothers had a lower obesity risk than those with US-born mothers [7]. Several studies by Chen et al. found an association between a lower maternal acculturation score on a multi-item questionnaire and elevated BMI in Chinese American children 8–10 years old [19,20,21,22]. These studies tended to be small and recruit from after-school programs and foreign-language schools in the San Francisco Bay Area [19,20,21,22], which may not represent the broader population.

### 1.2. Maternal Acculturation and Obesogenic Behaviors

Few studies directly assess the association between maternal acculturation and obesogenic behaviors in Asian American children [11,23]. Studies by Chen et al. found that a higher maternal acculturation score was associated with low sedentary activity [21], while both a lower maternal acculturation score and unhealthy food choices were risk factors for elevated BMI [22] in Chinese American children. Another study found that Chinese American parents (predominantly mothers) with a higher acculturation score tend to practice an indulgent feeding style, impose less restriction of unhealthy foods, and apply less pressure to eat healthy foods [24]. One study described the eating habits of Chinese American preschoolers whose caregivers had generally low acculturation scores but did not directly assess the relationship between caregiver acculturation and child diet [25]. Again, these studies tended to be small and/or recruit from after-school programs, foreign-language schools, and childcare centers, which may not be broadly representative. There is also a need to study the relationship between maternal acculturation and a wider range of obesogenic behaviors. 

This study examined the association between two proxies of maternal acculturation (nativity and time in the US), and obesity and several obesogenic behaviors in a relatively large sample of Asian American children in California. We assessed whether children whose mothers were born in the US or have been in the country longer are more likely to be obese and engage in obesogenic behaviors (low fruit and vegetable intake, high sugar-sweetened beverage and fast food intake, and high sitting activity).

## 2. Materials and Methods 

### 2.1. Data

This cross-sectional study examined public-use data from the California Health Interview Survey (CHIS), a continuous survey on various health topics [26]. The datasets generated and analyzed in this current study can be accessed on the CHIS website [26]. CHIS is the largest state health survey and provides representative data on all 58 counties in California [27]. CHIS uses a geographically stratified random-digit-dial telephone sampling strategy to randomly select households within counties and then one adult per household [28]. If present, children (birth to 11 years old) and adolescents (12–17 years old) may be interviewed separately [29]. Children are interviewed by proxy through the parent/guardian most knowledgeable about their health [29]. CHIS oversamples Korean and Vietnamese households [30] and conducts interviews in several languages [31] to increase Asian American representation. 

### 2.2. Sample

We pooled public-use data from the 2013–2016 CHIS for children 2–11 years old. These years were chosen to represent the most recently available datasets that include the variables of interest and define them similarly. We excluded children < 2 years because they have different BMI thresholds for overweight/obesity than older children [32]. Since obesity was a primary outcome, we excluded children with no reported weight. The data were otherwise complete for the variables of interest. To account for biologically implausible anthropometric data and reduce misclassification bias in BMI categorization due to parental misreporting, we excluded children with weights or heights beyond 1.5 times the interquartile range for age and sex. We included all remaining children that were reported as being non-Hispanic Asian (exclusive of mixed-race children), which we referred to as “Asian American”.

### 2.3. Measures

We used two proxies for maternal acculturation: nativity and time in the US. Maternal nativity was a binary variable: immigrant and US-born. Maternal time in the US was an ordinal variable with five levels: < 5 years, 5–14 years, 15–24 years, ≥ 25 years, and US-born. US nativity and increasing time in the US served as proxies for acculturation, similar to larger studies in Asian American populations [5,6,33,34,35,36] in which acculturation questionnaires were not administered.

BMI was organized into three categories based on age- and sex-specific guidelines from the Centers for Disease Control and Prevention [32]: not overweight/obese (BMI < 85th percentile), overweight (BMI 85th to < 95th percentile), and obese (BMI ≥ 95th percentile). In multivariate analyses exploring obesity as the outcome, children with BMI < 85th percentile served as the reference group. 

Dietary behaviors were dichotomized based on literature using CHIS data to examine Asian children’s obesogenic dietary practices [6]. Fruit and vegetable intake were determined by the number of caregiver-defined servings consumed yesterday. Given the low prevalence of soda and sweetened fruit drink consumption in the sample and the conceptual similarity of these drinks, they were combined into sugar-sweetened beverages (SSBs). SSB intake was determined by the number of glasses or cans of SSBs consumed yesterday. Fast food intake was defined as the number of times fast food was eaten in the past week. Similar to prior literature [6], consuming < 2 servings of fruit yesterday, < 2 servings of vegetables yesterday, ≥ 1 serving of SSB yesterday, and ≥ 1 serving of fast food last week were considered “obesogenic behaviors”.

Sedentary behavior was measured by sitting activity, defined as the number of hours spent sitting and watching TV, playing computer games, talking with friends, or doing other sitting activities on a typical weekday. Sitting activity was dichotomized, defining > 2 hours per day as an “obesogenic behavior” based on literature linking this amount of childhood TV time with adult overweight status [37].

Family income was identified as an important covariate and defined as a percentage of the federal poverty level (FPL): 0%–99% FPL, 100%–199% FPL, 200%–299% FPL, and ≥ 300% FPL. Besides child age and sex, there were no other relevant covariates, including ethnic subgroup and maternal education level, that were consistently available in the 2013–2016 public-use dataset.

### 2.4. Analyses

We computed descriptive statistics of the participants’ characteristics. Bivariate analyses were conducted using chi-square tests with the Rao–Scott second-order correction to account for the complex survey design. These analyses examined the associations between maternal nativity and time in the US, and child BMI and obesogenic behaviors. Separate multivariate logistic regression models examined the association between maternal nativity and time in the US, and child obesity and obesogenic behaviors, while adjusting for family income. Replicate weights provided by CHIS were used in all analyses to account for the complex survey design. All analyses were conducted using STATA/IC Version 15.1 (2019, StataCorp LLC, College Station, TX, USA), with statistical significance defined as *p* < 0.05.

### 2.5. Human Subjects

The University of California, Berkeley Institutional Review Board confirmed that this study was secondary analysis of a publicly available, de-identified database, which is not considered human subjects research. This study was conducted in accordance with the Declaration of Helsinki.

## 3. Results

### 3.1. Sample

Asian American children accounted for 8.4% of the 8507 children ages 2–11 years in the 2013–2016 CHIS dataset. From this subset, excluding those without a reported weight (n = 65) or an implausible weight/height (n = 39) yielded a sample of 609 children. 

### 3.2. Demographics

Table 1 displays the participants’ characteristics. Among the children, 55.4% were boys, and the mean age was 7 years. Most families (62.9%) were in the highest income bracket, and most mothers (76.2%) were foreign-born. Among immigrant mothers, 15.2% had been in the US for < 5 years, 36.6% for 5–14 years, 21.4% for 15–24 years, and 26.8% for ≥ 25 years.

### 3.3. Child BMI

Table 2 shows the children’s characteristics by BMI category and the results of related bivariate analyses. Overall, 9.6% of children were overweight, and 24.7% were obese. There was a significant association between income and BMI, with the lowest income group having more than 2.5 times the prevalence of obesity as the highest income group.

### 3.4. Maternal Nativity

In the multivariate analyses (Table 3), there was a trend toward children with US-born mothers having lower odds of obesity than those with immigrant mothers (*p* = 0.055), after adjusting for income. Higher income was significantly associated with lower odds of obesity. There were no associations between maternal nativity and obesogenic behaviors in either the bivariate or multivariate analyses. 

### 3.5. Maternal Time in the US

In the multivariate analyses (Table 3), children with US-born mothers had 88% lower odds of obesity than children with recent immigrant mothers (< 5 years in the US), after adjusting for income (*p* = 0.040). Higher income was significantly associated with lower odds of obesity.

In the bivariate analyses, there was a trend for the prevalence of low fruit intake (< 2 servings yesterday) to decrease with increasing maternal time in the US (*p* = 0.052). This finding was significant in the multivariate analyses (Table 4); compared to children of recent immigrants, children whose mothers had been in the US for ≥ 25 years had 89% lower odds of low fruit intake (*p* = 0.011), and children of US-born mothers had similarly lower odds (*p* = 0.027), after adjusting for income. There were no associations between maternal time in the US and other obesogenic behaviors.

In the multivariate analyses, children whose mothers had been in the US for 5–14, 15–24, or ≥ 25 years had similar odds of obesity (Table 3) and low fruit intake (Table 4), which were lower than for children of recent immigrants. We performed post-hoc analyses to examine whether these three groups could be considered one homogenous group. We re-coded maternal time in the US into three levels: < 5 years, ≥ 5 years, and US-born. The odds of obesity and low fruit intake for the combined ≥ 5 year group were essentially unchanged and remained lower than for the < 5 year group, though only the difference in fruit intake was significant. Specifically, children whose mothers have been in the US for ≥ 5 years had lower odds of low fruit intake (adjusted odds ratio, AOR, 0.19; 95% CI 0.04 to 0.90) than children of recent immigrants, after adjusting for income.

## 4. Discussion

This study explored the relationship between maternal nativity and time in the US, and child obesity and obesogenic behaviors in Asian Americans. Children with US-born mothers were not more likely to be obese than those with immigrant mothers as a whole. This finding differs from a study showing that Asian American preschool-age children with immigrant mothers are less likely to be obese than those with US-born mothers [7]. The contrast may be due to mothers being particularly influential on the diet [15] and physical activity [18] of younger children. The older children included in our study might be more influenced by external factors that attenuate any protective effects of having immigrant mothers.

The likelihood of obesity did not increase with increasing maternal time in the US. The only significant difference in the odds of obesity was between children of recent immigrants (< 5 years in the US) and children with US-born mothers. Using less time in the US as a proxy for lower acculturation, this result strengthens several studies in Chinese American children linking lower maternal acculturation scores with elevated BMI [19,20,21,22]. This study highlights children of recent immigrants as a vulnerable group that should be addressed in future obesity prevention research and interventions and challenges the notion that lower acculturation is protective. While prior studies have shown Asian American children as a whole to have a relatively low risk of elevated BMI [1,2,3], this study adds nuance to the literature by suggesting that the risk of obesity is not homogenous in this population. Specifically, a subgroup of children with recent immigrant mothers have a higher risk of obesity than peers with US-born mothers. It is unclear whether children of recent immigrants tend to arrive in the US already obese or adopt obesogenic behaviors as they are exposed to a Westernized environment. Children of recent immigrants may also tend to come from countries where they experience food insecurity and encounter dietary excess upon arrival in the US [38]. This abrupt change in dietary environment may lead to an initially higher risk of obesity, which may stabilize or decrease as they spend more time in the US. These processes are important to explore further.

Few studies directly assess the relationship between maternal acculturation and obesogenic behaviors in Asian American children [11,23], and this study adds to the literature by exploring several behaviors. Children whose mothers were US-born or have been in the country longer were not more likely to engage in obesogenic behaviors. Children of recent immigrants were actually more likely to have low fruit intake than those whose mothers were US-born or have been in the US for ≥ 25 years. This finding aligns with literature suggesting that maternal acculturation explains differences in fruit consumption among Asian ethnic groups [6].

In post-hoc analyses, children whose mothers have been in the US for ≥ 5 years were less likely to have low fruit intake than children of recent immigrants. This finding adds complexity to the literature by highlighting the heterogeneity in dietary behavior among Asian American children and specifically identifying children of recent immigrants as a nutritionally vulnerable subgroup. Recent immigrants may consider fruit a special occasion item that is less important than other foods [39]. Culturally preferred fruits may also be more costly or difficult to find [40]. Increasing access to affordable, culturally acceptable fruits may improve intake in children of recent immigrants.

There are several reasons why children whose mothers are US-born or have been in the country longer may not be more likely to engage in obesogenic behaviors. With the rise of caloric processed foods in Asian countries due to globalization and urbanization [41,42], recent immigrants may already have obesogenic behaviors upon arrival and have a similar or higher risk of obesity than US-born peers. Additionally, increased time in the US may not equate to increased adoption of Western behaviors. Recent immigrants may come from countries with a heavy Western influence and already have obesogenic practices before arriving. Conversely, mothers who have lived in the US for decades may maintain a traditional diet and expose their children to these foods. Finally, maternal and child diets may differ [23,38,43], despite parental efforts to retain a traditional diet [38,43], particularly in older children.

Lower income was consistently associated with a higher odds of obesity. Adjusting for income attenuated the initially significant difference in the odds of obesity between children of immigrant versus US-born mothers. This finding reinforces the link between low income and elevated BMI [4] and the interconnectedness of socioeconomic status and acculturation [5] in Asian American children. While Asian Americans are the highest earning racial/ethnic group, they are the most economically divided, partly due to different sociopolitical histories and immigration policies that shape their experiences [44]. Furthermore, low acculturation is associated with food insecurity in most Asian American subgroups [35], and participation in the Supplemental Nutrition Assistance Program (SNAP) is low in this population [35]. Providing financial assistance to low income, less acculturated families and addressing barriers to SNAP participation may help reduce obesity in Asian American children. Community-based interventions that are sensitive to cultural food preferences can also be helpful in promoting healthier dietary habits in Asian American populations [45].

This study has several strengths. We used a dataset designed to be representative of a state with a large Asian American population, with oversampling of less represented subgroups. The sample was larger than many studies exploring the relationship between maternal acculturation and obesogenic behaviors in Asian American children. We examined two proxies for acculturation, which are important to distinguish, and several obesogenic behaviors.

In terms of limitations, causal relationships cannot be established in this cross-sectional study. Caregiver-reported data may reduce internal validity, including in BMI classification [46], though we did attempt to reduce misclassification bias by omitting biologically implausible data. Additionally, asking caregivers to describe children’s fruit, vegetable, or SSB intake over the past day may not capture their typical diet. Other dietary assessment methods, such as 7-day food records, may be more representative but potentially more burdensome. We were unable to conduct ethnic subgroup analysis because this information was not consistently available in the public-use CHIS dataset for children in 2013–2016. Studies have noted interethnic variation in the risk of child obesity and obesogenic behaviors [4,5,6,7]. Thus, care should be taken in extrapolating our findings to a particular subgroup, and further studies are needed to assess the replicability of our results in different Asian American ethnicities. Given the limitations of the dataset, we were also unable to examine the effects of maternal education level, which has previously been described as an important factor in the relationship between maternal acculturation and child BMI [5]. Additionally, we used proxies that do not fully capture the concept of acculturation. Acculturation is a complex process that affects beliefs, attitudes, and behaviors [21] and can manifest in multiple dimensions of life [20] in varying ways. Smaller studies have used multi-item acculturation questionnaires [19,20,21,22,25], but nativity and time in the US have been used as proxies in larger studies in Asian American populations in which detailed acculturation scales were not administered [5,6,33,34,35,36]. Finally, we used data from 2013-2016, which may not be representative of the current population. However, there is a lag in the availability of yearly data in the public-use CHIS dataset, and we needed to pool multiple years given the small number of Asian American children. This limitation speaks to the need for larger datasets on the health of Asian American children for future research.

## 5. Conclusions

While Asian American children are known to have a relatively low risk of elevated BMI, a subgroup of children with recent immigrant mothers are more likely to be obese and eat less fruit than those with US-born mothers. Children of immigrants are not homogeneous, and efforts to prevent obesity and increase fruit consumption are particularly important for a more vulnerable population of Asian American children of recent immigrants.

## Figures and Tables

**Table 1:** Weighted characteristics of Asian American children ages 2 to 11 years old and their mothers (n = 609): California Health Interview Survey (CHIS) 2013–2016.

Child Age, Mean Years (sd ^1^)	7.0 (2.8)
Child sex, %	
Male	55.4
Female	44.6
Family income, %	
0%–99% FPL^2^	12.5
100%–199% FPL	16.2
200%–299% FPL	8.4
≥300% FPL	62.9
Maternal nativity, %	
Immigrant	76.2
US-born	23.8
Maternal time in US, %	
<5 years	11.6
5–14 years	27.9
15–24 years	16.3
≥25 years	20.4
US-born	23.8
Child BMI category, %	
Not overweight	65.7
Overweight	9.6
Obese	24.7
Obesogenic behaviors, %	
<2 servings of fruit yesterday	49.0
<2 servings of vegetables yesterday	55.8
≥1 serving of SSB^3^ yesterday	28.9
≥1 serving of fast food last week	64.9
>2 sitting activity hours per day	28.1

^1^ standard deviation, ^2^ federal poverty level, ^3^ sugar-sweetened beverage.

**Table 2:** Weighted characteristics of Asian American children ages 2 to 11 years old by BMI category (n = 609): CHIS 2013-2016.

	Not OW/OB ^3^	Overweight	Obese	*p*-Value
Children, %	65.7	9.6	24.7	—
Child age, mean years (sd ^1^)	7.1 (2.8)	8.2 (3.0)	6.3 (2.4)	—
Child sex, %				0.154
Male	63.9	6.0	30.2	
Female	67.9	14.1	17.9	
Family income, %				0.018 *
0%–99% FPL ^2^	37.7	12.7	49.6	
100%–199% FPL	49.6	6.8	43.7	
200%–299% FPL	89.9	7.2	2.9	
≥ 300% FPL	72.1	10.1	17.8	
Maternal nativity, %				0.066
Immigrant	60.9	10.0	29.1	
US-born	81.1	8.4	10.6	
Maternal time in US, %				0.155
< 5 years	33.9	9.3	56.8	
5–14 years	62.8	10.1	27.1	
15–24 years	69.0	7.7	23.3	
≥ 25 years	67.1	12.1	20.8	
US-born	81.1	8.4	10.6	

^1^ standard deviation, ^2^ federal poverty level, ^3^ overweight/obese, * *p* < 0.05.

**Table 3:** Association between maternal nativity and maternal time in the US and obesity among Asian American children ages 2 to 11 years old (n = 609): CHIS 2013-2016.

	OR ^3^ (95% CI ^5^)	*p*-Value	Overall *p*-Value	AOR^4^ (95% CI)	*p*-Value	Overall *p*-Value
Maternal nativity			—			—
Immigrant (ref ^1^)	1.00	—		1.00	—	
US-born	0.27 (0.09–0.81)	0.020 *		0.31 (0.10–1.02)	0.055	
Family income						0.001 *
0%–99% FPL ^2^ (ref)				1.00	—	
100%–199% FPL				0.69 (0.11–4.54)	0.700	
200%–299% FPL				0.03 (0.00–0.25)	0.001*	
≥300% FPL				0.20 (0.04–1.05)	0.058	
	OR (95% CI)	*p*-value	overall *p*-value	AOR (95% CI)	*p*-value	overall *p*-value
Maternal time in US			0.054			0.267
< 5 years (ref)	1.00	—		1.00	—	
5–14 years	0.26 (0.05–1.30)	0.100		0.34 (0.05–2.28)	0.267	
15–24 years	0.20 (0.04–1.12)	0.067		0.34 (0.04–2.72)	0.307	
≥ 25 years	0.19 (0.02–1.56)	0.121		0.27 (0.02–2.96)	0.282	
US-born	0.08 (0.01–0.41)	0.003 *		0.12 (0.02–0.91)	0.040 *	
Family income						0.008 *
0%–99% FPL (ref)				1.00	—	
100%–199% FPL				0.67 (0.07–6.55)	0.727	
200%–299% FPL				0.03 (0.00–0.39)	0.007 *	
≥ 300% FPL				0.24 (0.03–2.13)	0.201	

^1^ reference group, ^2^ federal poverty level, ^3^ odds ratio, ^4^ adjusted odds ratio (controlling for family income), ^5^ confidence interval, * *p* < 0.05.

**Table 4:** Association between maternal time in the US and obesogenic behaviors among Asian American children ages 2 to 11 years old (n = 609): CHIS 2013-2016.

	< 2 Servings of Fruit Yesterday		< 2 Servings of Vegetables Yesterday		≥ 1 Serving of SSB^4^ Yesterday		≥ 1 Serving of Fast Food Last Week		> 2 Sitting Activity Hours Per Day	
	AOR^5^ (95% CI^6^)	*p ^7^*	AOR (95% CI)	*p*	AOR (95% CI)	*p*	AOR (95% CI)	*p*	AOR (95% CI)	*p*
Mat^1^ time in US										
< 5 years (ref ^3^)	1.00	—	1.00	—	1.00	—	1.00	—	1.00	—
5–14 years	0.25 (0.05–1.25)	0.091	0.36 (0.06–2.21)	0.269	5.50 (0.78–38.64)	0.086	0.78 (0.13–4.53)	0.782	0.33 (0.06–1.92)	0.218
15–24 years	0.21 (0.04–1.14)	0.071	0.76 (0.12–4.71)	0.766	6.07 (0.85–43.22)	0.071	0.96 (0.15–6.06)	0.964	0.12 (0.02–0.80)	0.028 *
≥ 25 years	0.11 (0.02–0.60)	0.011 *	0.56 (0.08–3.80)	0.553	4.72 (0.49–45.13)	0.178	0.97 (0.13–6.96)	0.973	0.53 (0.07–4.24)	0.549
US-born	0.15 (0.03–0.81)	0.027 *	0.52 (0.08–3.37)	0.489	2.10 (0.26–17.19)	0.489	0.97 (0.15–6.39)	0.979	0.61 (0.10–3.83)	0.594
Overall *p* (ob ^2^ behaviors)	0.111		0.672		0.213		0.995		0.165	
Overall *p* (family income)	0.999		0.343		0.679		0.712		0.875	

^1^ maternal, ^2^ obesogenic, ^3^ reference group, ^4^ sugar-sweetened beverage, ^5^ adjusted odds ratio (controlling for family income), ^6^ confidence interval, ^7^ *p*-Value, * *p* < 0.05.
